# Prognostic Value of a Histopathological Scoring System and the Ki67 Proliferation Index in Dogs With Phaeochromocytoma

**DOI:** 10.1111/vco.70021

**Published:** 2025-09-03

**Authors:** Marit F. van den Berg, Aitor Martínez Ruiz, Maurice M. J. M. Zandvliet, Sebastiaan A. van Nimwegen, Hans S. Kooistra, Sara Galac, Guy C. M. Grinwis

**Affiliations:** ^1^ Department Clinical Sciences, Faculty of Veterinary Medicine Utrecht University Utrecht the Netherlands; ^2^ Department Biomolecular Health Sciences, Faculty of Veterinary Medicine Utrecht University Utrecht the Netherlands

**Keywords:** adrenal tumour, canine, GAPP, PASS, survival

## Abstract

Canine phaeochromocytomas (PCCs) are neuroendocrine tumours with malignant potential. Metastatic disease remains the sole definitive evidence of malignancy. Histopathological criteria to predict long‐term survival have not been established in dogs. This study evaluated the reproducibility and prognostic value of histopathological parameters derived from human scoring systems, along with the Ki67 proliferation index (PI), in dogs after adrenalectomy for PCC. Tumour samples from 41 dogs were assessed by a veterinary pathologist and pathology resident. Of 10 histopathological parameters examined, only necrosis, tumour cell spindling, and extension into adipose tissue achieved sufficient inter‐ and intra‐observer agreement (≥ 0.40) for inclusion in survival analyses, while Ki67 PI demonstrated excellent reproducibility (≥ 0.95). A composite histopathological score was generated by summing these three parameters and a dichotomised Ki67 PI (optimal cutoff 18%), as determined by ROC analysis. Among the 41 dogs, eight died within 2 weeks postoperatively, leaving 33 long‐term survivors with four tumour‐related events. Kaplan–Meier analysis showed significantly poorer survival (*p* < 0.001) in dogs with a high Ki67 PI (≥ 18%), whereas the composite score showed a borderline significant association with outcome in Cox regression (*p* = 0.056; hazard ratio 2.80). Overall, dogs surviving the immediate postoperative period demonstrated a favourable prognosis (mean overall survival of 2456 days). These findings suggest that, in this cohort with few tumour‐related events, the dichotomised Ki67 PI alone may serve as a clinically applicable prognosticator for canine PCC. However, further research in larger populations is needed to determine whether a composite score adds prognostic value and guides postoperative management.

**Abbreviations**
ADH: adrenal‐dependent hypercortisolism
CHGA: chromogranin A
CI: confidence interval
GAPP: Grading of AdrenalPhaeochromocytoma and Paraganglioma
HR: hazard ratio
IHC: immunohistochemistry
Ki67 PI: Ki67 proliferation index
PASS: Phaeochromocytoma of the Adrenal Gland Scaled Score
PCC: phaeochromocytoma
ROC: receiver operating characteristic
SDHB: succinate dehydrogenase subunit B
SYP: synaptophysin
UCCR: urinary corticoid: creatinine ratio

## 1 Introduction

Phaeochromocytomas (PCCs) are neuroendocrine tumours that originate from chromaffin cells in the adrenal medulla, affecting both humans and dogs. Historically, PCCs in dogs were classified as malignant based on the presence of local invasion or metastasis [1]. Although local invasion, including vascular invasion, is commonly observed in dogs, with reported frequencies ranging from 33% to 82% [2, 3, 4, 5], it is no longer considered a definitive indicator of malignancy in human PCCs [6]. In contrast, metastatic disease, present in 13%–24% of cases at diagnosis in dogs [7, 8], is the only widely accepted evidence of malignancy [6]. In humans, the 2022 World Health Organization classification deems all PCCs to have malignant potential [6]—a notion that is likely applicable to canine PCCs as well. When feasible, adrenalectomy is the treatment of choice for canine PCC. However, even among those dogs that survive the perioperative period and often experience prolonged survival [4, 5, 9], there is a risk of recurrent disease and PCC‐related metastasis—mirroring findings in humans, where the development of metastatic disease after surgery reduces life expectancy [10, 11, 12].

In human medicine, established histopathological scoring systems, such as the Phaeochromocytoma of the Adrenal Gland Scaled Score (PASS [13]) and the Grading of Adrenal Phaeochromocytoma and Paraganglioma (GAPP [14]), are employed to evaluate malignant potential. Notably, subsequent studies have demonstrated that grading of the histological parameters included in PASS exhibits high inter‐ and intra‐observer variation and only low‐to‐moderate interrater reliability, even when applied by pathologists with extensive experience in endocrine pathology [15, 16]. Comparable validated systems for dogs are currently lacking. In a study by Zini et al. (2019) [1], PASS and various immunohistochemical markers were assessed in dogs with PCC to evaluate their correlation with surgical outcomes, but they could not differentiate between dogs that survived to discharge and those that died post‐adrenalectomy. Moreover, no studies have investigated the relationship between these scoring systems and long‐term survival in dogs.

This retrospective study had two main objectives. First, we aimed to assess whether histopathological parameters—derived from the human scoring systems PASS and GAPP—demonstrate sufficient inter‐ and intra‐observer reliability when applied to canine PCCs. Second, we sought to develop a histopathological scoring system for canine PCCs using these reliable parameters, evaluating both individual parameters and the predictive value of their composite score for long‐term outcomes following adrenalectomy.

## 2 Methods

### 2.1 Population and Sample Collection

Canine PCCs were collected between 2012 and 2024 following adrenalectomy. Samples from 16 dogs were collected at the Department Clinical Sciences, Faculty of Veterinary Medicine, Utrecht University. In addition, samples were collected from contributing veterinary institutions, including University of Bologna (*n* = 7), Ghent University (*n* = 6), University of Ljubljana (*n* = 1), and veterinary referral centres in the Netherlands (*n* = 6), Italy (*n* = 2), Slovenia (*n* = 2), and Sweden (*n* = 1). Dogs were excluded if metastatic disease was identified before or during surgery, based on preoperative diagnostic imaging (typically thoracoabdominal CT, or abdominal ultrasound and thoracic radiographs) and, if available, cytological or histopathological evaluation of suspicious lesions, or if they had less than 3 months of follow‐up data. Although we recorded outcomes for dogs that died within 2 weeks postoperatively, these short‐term postoperative deaths were excluded from the survival analyses to minimise the impact of surgery or anaesthesia‐related complications on outcome. To confirm the diagnosis of PCC, immunohistochemistry (IHC) for chromogranin A (CHGA) and synaptophysin (SYP) was performed as previously described [17], with all tumours confirmed as PCC based on the positivity of at least one marker. Only dogs with a definitive histopathological diagnosis of PCC were included, regardless of their plasma/urinary metanephrine concentrations.

### 2.2 Clinical Parameters

Medical records were retrospectively reviewed to gather information on breed, sex/neutering status, age at surgery, clinical signs, biochemical findings (e.g., plasma/urinary metanephrines), imaging results, tumour laterality (left, right, or bilateral), presence of vascular invasion, tumour size, presence of metastasis, and follow‐up. All cytologic and histologic findings from suspected metastatic lesions or other tissues, including IHC results, were also recorded. Tumour size was recorded as the maximum diameter based on diagnostic imaging (CT, or ultrasound if CT was unavailable), as reported in the radiology report. PCCs were deemed biochemically negative when plasma/urinary metanephrines did not exceed the published upper reference limits for healthy dogs [18, 19].

### 2.3 Histopathological Parameters

For histological evaluation, a representative portion of each neoplasm was fixed in 4% buffered formaldehyde for histopathology and immunohistochemistry and subsequently submitted to the pathology laboratory. The tissues were routinely embedded in paraffin and cut into 4‐μm sections. Tissue sections for each PCC were stained with haematoxylin and eosin. Glass slides were manually evaluated using a light microscope. Areas of the neoplasms containing artefacts arising from sampling, tissue handling, or processing were excluded from analysis. Ten histopathological parameters were evaluated for each tumour: growth pattern, necrosis, cellularity, cellular monotony, tumour cell spindling, atypical mitotic figures, extension into adipose tissue, vascular invasion, capsular invasion, and nuclear pleomorphism. These parameters were selected based on the histopathological features included in the PASS and GAPP grading schemes [13, 14]. All parameters were assessed independently by one veterinary pathologist experienced in neuroendocrine pathology (G.G.) and one resident in veterinary pathology (A.M.R.), with both observers blinded to the clinical outcomes. Each parameter was evaluated twice by each observer, with a minimum interval of 2 weeks between the two assessments.

**FIGURE 1:**
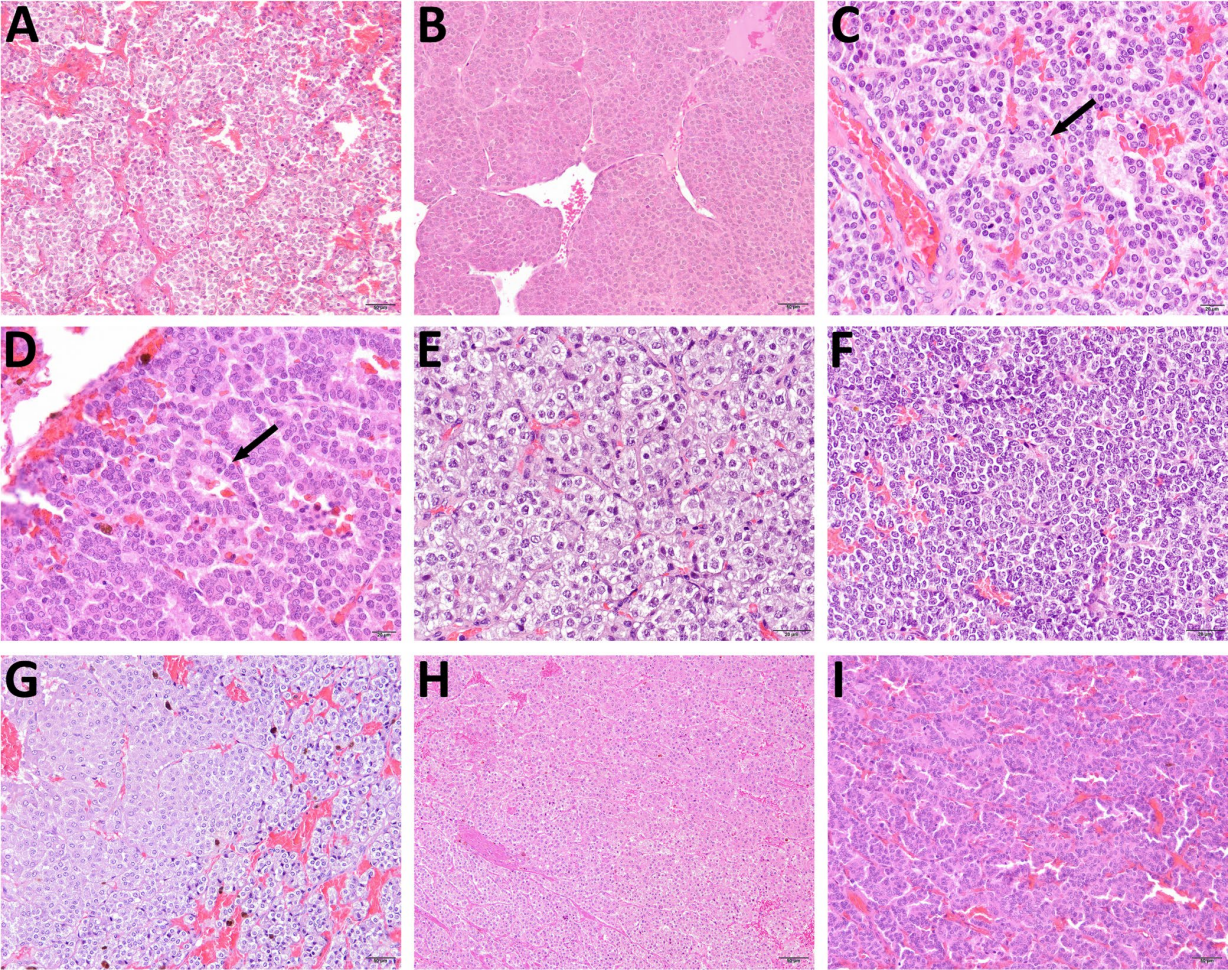
Representative histopathological images illustrating the scoring criteria for growth pattern (A–D), cellularity (E, F), and cellular monotony (G–I). Haematoxylin and eosin stain. (A) Growth pattern score 0: Regular cell nests with uniform shape and size, showing only little variation. (B) Growth pattern score 1: Irregular cell nests with variable shapes and sizes, including nests up to 10 times larger than the smallest ones. Growth pattern score 2: Presence of rosettes (arrow; C) or pseudorosettes (arrow; D). (E) Cellularity score 1: Moderate cellularity, with neoplastic cells showing a moderate to abundant amount of cytoplasm and a medium‐sized, round nucleus. (F) Cellularity score 2: High cellularity, with neoplastic cells showing a moderate to scant amount of cytoplasm and a centrally located small nucleus. (G) Monotony score 0: Low monotony, neoplastic cells with two or more distinguishable cytoplasmic morphologies, for example, one with a moderate amount of pale amphophilic, uniform to slightly granular cytoplasm (left), and another with smaller cells showing moderate to scant, highly vacuolated cytoplasm (right). (H) Monotony score 1: Moderate monotony, neoplastic cells of similar size with slight cytoplasmic differences, ranging from a moderate amount of eosinophilic, uniform cytoplasm (top right), to slightly vacuolated or granular, paler cytoplasm (bottom left). (I) Monotony score 2: High monotony, neoplastic cells with uniform size, shape, and cell borders, and similar cytoplasmic appearance.

**FIGURE 2:**
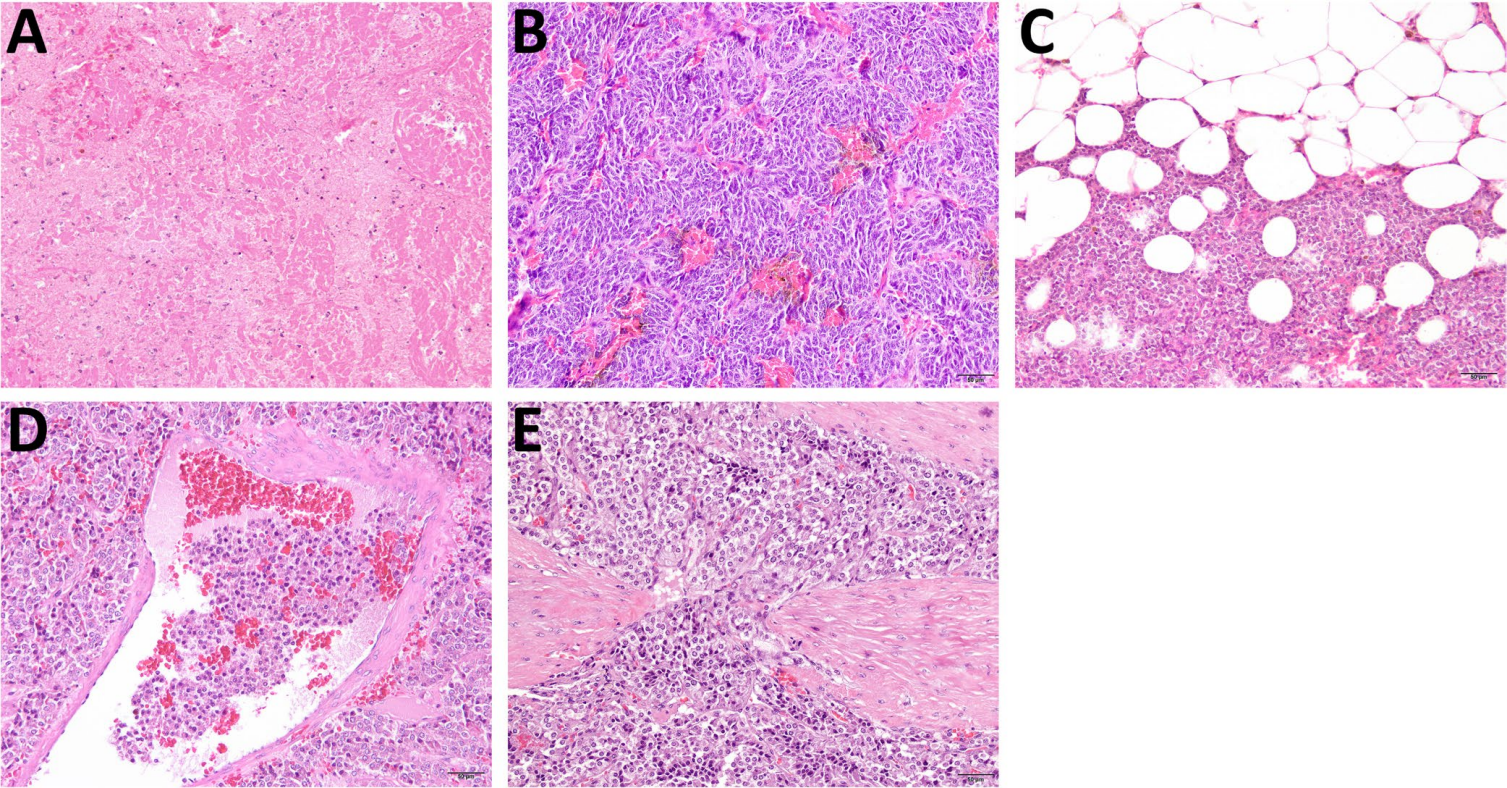
Representative histopathological images illustrating necrosis (A), tumour cell spindling (B), extension into periadrenal adipose tissue (C), vascular invasion (D), and capsular invasion (E). Haematoxylin and eosin stain. (A) Necrosis: Focal, extensive area where the neoplastic architecture has been replaced by amorphous eosinophilic material containing occasional cells with hypereosinophilic cytoplasm and shrunken, hyperchromatic nuclei (karyopyknosis), fragmented nuclei (karyorrhexis), or nuclear loss (karyolysis). (B) Tumour cell spindling: Neoplastic cells with typical spindle‐shaped morphology. (C) Extension into adipose tissue: Groups of neoplastic cells invading the periadrenal adipose tissue. (D) Vascular invasion: A group of neoplastic cells within a blood vessel lumen. (E) Capsular invasion: Neoplastic cells invading a fibrous capsule.

**FIGURE 3:**
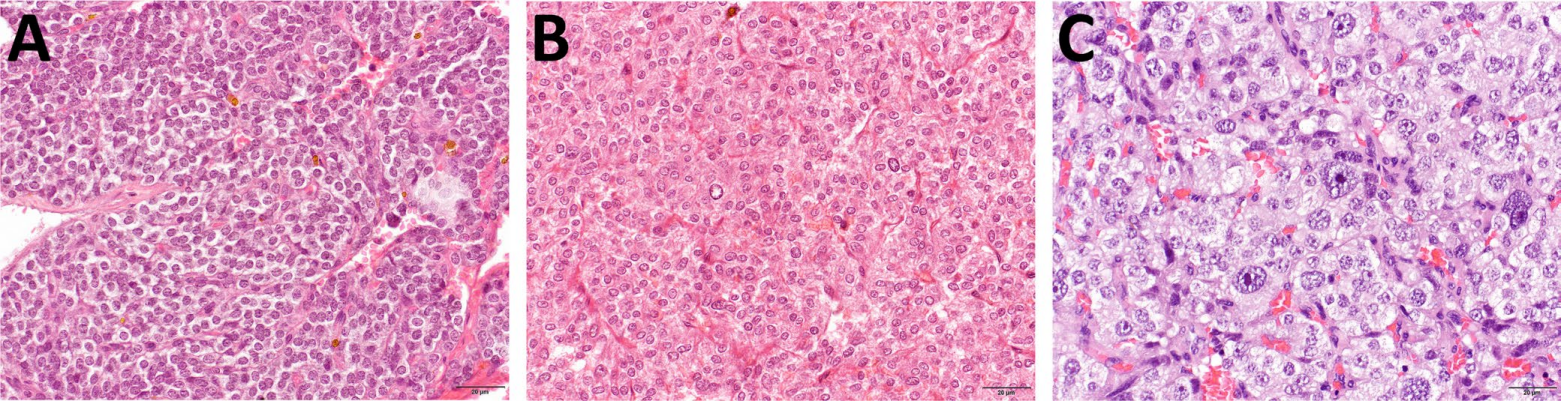
Representative histopathological images illustrating the scoring criteria for nuclear pleomorphism. Haematoxylin and eosin stain. (A) Mild nuclear pleomorphism (score 0): Neoplastic cells show uniform nuclear morphology and size, with indistinct or one distinct nucleolus and finely stippled chromatin. (B) Moderate nuclear pleomorphism (score 1): Neoplastic cells show frequent mild to moderate variation in nuclear shape and size, with the largest nuclei up to three times the size of the smallest ones, and occasionally with up to two nucleoli per nucleus. (C) Marked nuclear pleomorphism (score 2): Neoplastic cells show large nuclei (karyomegaly), occasionally with irregular shapes and up to three prominent nucleoli.

A scoring system was developed for each parameter, using a qualitative approach for growth pattern, necrosis, tumour cell spindling, atypical mitotic figures, extension into adipose tissue, vascular invasion, and capsular invasion, and a semiquantitative approach for cellularity, cellular monotony, and nuclear pleomorphism. For each parameter, a numeric value was assigned: 0 or 1 for qualitative parameters based on absence/presence, and 0, 1, or 2 for the semiquantitative parameters and growth pattern (Table 1). The scoring criteria and corresponding values for each parameter were established as follows: for growth pattern, neoplastic cell nests were categorised into three groups based on size, shape, and architecture. Regular cell nests with uniform size and shape were assigned a score of 0, the presence of irregular cell nests (with nests 10 times larger than the smallest ones, even if focal) was scored as 1, and the presence of rosettes or pseudorosettes, even if focal, was scored as 2 (Figure 1A–D). When multiple scoring features were present within a single tumour, the highest applicable score was assigned. Cellularity was classified as low, moderate, or high according to the number of neoplastic cells per field of vision, avoiding areas of stroma, necrosis, fibrosis, or haemorrhage (Figure 1E,F). Cellular monotony was evaluated by assessing differences in cellular size, shape, border distinction, and cytoplasmic appearance (homogeneous, vacuolated, or granular) and was scored as low, moderate, or high (Figure 1H–J). Nuclear morphology was assessed separately. Necrosis was scored based on the presence of areas where neoplastic cells exhibited morphological changes compatible with pyknosis, karyorrhexis, or karyolysis (Figure 2A). Tumour cell spindling was determined by the presence of a cluster of neoplastic cells with a characteristic elongated or spindle cell morphology, even if focal (Figure 2B). Extension into adipose tissue was scored based on the presence of groups of neoplastic cells invading and expanding the spaces between adipocytes in the periadrenal adipose tissue (Figure 2C). Vascular invasion was scored based on the presence of neoplastic cells within the lumen of a blood or lymphatic vessel (neoplastic emboli; Figure 2D). Capsular invasion was scored based on the invasion of the fibrous capsule by tumour cells, even if only focal (Figure 2E). Atypical mitotic figures were scored based on the presence of mitoses with features such as bipolar or multipolar asymmetry, segregation abnormalities, or irregular/amorphous chromatin distribution. Finally, nuclear pleomorphism was assessed based on nuclear size, shape, chromatin distribution, and the number of nucleoli. The most defining feature for this parameter was variation in nuclear size. Tumours received a score of 0 when the nuclei demonstrated regular size and shape, finely stippled chromatin, and an indistinct or single centrally located nucleolus. A score of 1 was assigned when any of the following features were observed: nuclei up to three times larger than the smallest ones, irregular nuclear shape, uneven chromatin distribution, or up to two nucleoli per nucleus. A score of 2 was given in two situations: (1) when karyomegaly (i.e., nuclei more than three times larger than the smallest ones) was present, or (2) when nuclear size variation was less pronounced but was accompanied by additional features such as binucleation, irregular nuclear shapes, uneven chromatin distribution, or more than three nucleoli per nucleus (Figure 3). For both scores 1 and 2, the relevant features had to be observed across ≥ 4 high‐power fields (field of vision of 0.34 mm^2^), rather than as isolated or rare findings.

**TABLE 1:** Scoring scheme for histopathological parameters.

Parameters	Categories	Score if present
Growth pattern	Regular cell nests	0
	Irregular cell nests with nests at least 10 times larger than smaller ones (even focal)	1
	(Pseudo)rosettes (even focal)	2
Necrosis	Absent	0
	Present	1
Cellularity	Low	0
	Moderate	1
	High	2
Cellular monotony	Low	0
	Moderate	1
	High	2
Tumour cell spindling	Absent	0
	Present	1
Atypical mitotic figure(s)	Absent	0
	Present	1
Extension into periadrenal adipose tissue	Absent	0
	Present	1
Vascular invasion	Absent	0
	Present	1
Capsular invasion	Absent	0
	Present	1
Nuclear pleomorphism	Mild	0
	Moderate	1
	Marked	2

*Note*: Scoring scheme for the ten histopathological parameters evaluated in canine phaeochromocytomas.

### 2.4 Ki67 Proliferation Index

Immunohistochemistry for Ki67 proliferation index (PI) was performed as previously described [20], using a mouse monoclonal primary anti‐Ki67 antibody (Agilent Cat# M7240, RRID: AB_2142367). Positive controls consisted of normal canine colon tissue slides, while negative controls were prepared by omitting the primary antibody. Before counting, the slides were assessed to identify “hot spot regions” with positive PCC cells, capturing at least 2–3 photographic images for each case, at 200× magnification. Two observers (M.v.d.B. and A.M.R.) then manually counted the percentage of neoplastic cells showing nuclear positivity in at least 1000 cells using ImageJ software [21]. Areas suspected of extramedullary haematopoiesis were excluded from analysis. The Ki67 PI score was calculated as the proportion of Ki67‐positive nuclei compared to the total nuclei counted for each PCC. One observer (A.M.R.) assessed each tumour once, while the other observer (M.v.d.B.) performed Ki67 scoring twice for 19 tumours to assess intra‐observer agreement.

### 2.5 Statistical Analyses

Statistical analyses were performed using IBM SPSS Statistics for Windows, version 29 (IBM Corp., Armonk, NY).

#### 2.5.1 Observer Agreement

The intra‐ and inter‐observer agreement scores for categorical variables were calculated using the Cohen's kappa coefficient, while the intra‐class correlation coefficient was used for Ki67 PI (continuous variable). Agreement strength was classified as < 0.40 (poor), 0.40–0.59 (moderate), 0.60–0.79 (good), and 0.80–1.00 (excellent) [20]. Inter‐observer comparisons were performed by matching each observer's first scoring session (score 1) to the other observer's first session, and each observer's second session (score 2) to the other's second session. Only parameters with agreement scores ≥ 0.40 for both intra‐observer (each observer) and inter‐observer assessments were included in the survival analyses. For categorical variables, the most frequent result (among observers) was recorded. In cases of a tie, a consensus decision was reached through discussion. For continuous variables, the average of the two observers' measurements was used.

#### 2.5.2 Receiver Operating Characteristic (ROC) Analysis

Based on ROC curve data, Youden's index was used to identify optimal cutoff points for continuous measures (Ki67 PI). The chosen cutoffs were then treated as binary variables in subsequent analyses.

#### 2.5.3 Composite Histopathological Score

A composite histopathological score was generated by summing the scores of individual histopathological parameters that met the reliability criteria, along with the binary score for Ki67 PI. This composite score was then analysed as a continuous variable in relation to long‐term outcomes.

#### 2.5.4 Survival Analysis

An event was defined as death or euthanasia attributable to PCC, based on diagnostic evidence of tumour recurrence or metastasis (i.e., imaging‐confirmed local regrowth or distant spread), in the absence of indications for other neoplastic processes. Additionally, any death for which a PCC‐related cause could not be confidently excluded was also classified as an event. Dogs that died from unrelated causes were lost to follow‐up or remained alive at the study's end were censored at their last known survival time. Kaplan–Meier product‐limit estimation was used to calculate survival times, and the log‐rank test compared survival distributions for categorical variables. Cox proportional hazards regression was applied to continuous variables. Variables with *p* ≤ 0.20 in univariate analysis were considered for a multivariate Cox model, and *p* < 0.05 was deemed significant. Where applicable, hazard ratios (HR) and 95% confidence intervals (CI) are reported. In addition to the histopathological parameters and Ki67 PI, tumour size (maximum diameter based on diagnostic imaging) was evaluated in Cox regression to assess potential prognostic value. The reverse Kaplan–Meier method (treating an event as a censure and a censure as an event) was used to estimate the median follow‐up time.

## 3 Results

### 3.1 Population

Tumour tissue was collected from 41 client‐owned dogs diagnosed with PCC and treated via adrenalectomy. Of these, 26 were male (17 neutered, 9 intact) and 15 were female (all neutered). The median age at the time of surgery was 11.3 years (range, 7.1–14.0 years). Breeds included nine mixed breeds, three Jack Russell Terriers, three Galgo Españoles, two Pugs, two Rhodesian Ridgebacks, two Poodles, two Chihuahuas, two Border Terriers, two Kooikers, and one each of Shih Tzu, Vizsla, Labradoodle, Australian Labradoodle, Stabyhoun, Cavalier King Charles Spaniel, Staffordshire Bull Terrier, Dachshund, Maltese, Welsh Springer Spaniel, Chinese crested dog, Irish Setter, Australian cattle dog, and Cane Corso.

### 3.2 Clinical Parameters

Adrenalectomy was performed for a unilateral adrenal mass in 39 dogs (24 left‐sided, 11 right‐sided, and 4 side unspecified) and for bilateral disease in 2 dogs. Median tumour size was 2.6 cm (range, 0.8–7.1 cm) in maximal dimension. Macroscopic vascular invasion on imaging was reported in 28 dogs, involving the caudal vena cava (*n* = 12), phrenicoabdominal vein (*n* = 8), both vessels (*n* = 4), caudal vena cava and renal vein (*n* = 1), renal and portal vein (*n* = 1), or unspecified vessels (*n* = 2). In 11 dogs, no vascular invasion was reported, and in two cases, this information was not available. Thirty dogs had increased metanephrine concentrations (*n* = 29 in plasma, *n* = 1 in urine), whereas four dogs had biochemically negative PCC. In two of these four cases, an adrenal mass was discovered incidentally during diagnostic imaging without prior evidence of adrenal disease, and in one dog, an adrenal mass was identified during workup for hypertension, weakness, exercise intolerance, vomiting, and diarrhoea. In the remaining case, endocrine evaluation performed in the context of an adrenal mass led to a diagnosis of adrenal‐dependent hypercortisolism (ADH); a concurrent PCC was only diagnosed during histopathological examination, where an adrenocortical tumour was also identified. Additionally, in seven dogs, metanephrine levels were not determined.

It is also worth noting that, following adrenalectomy, three other dogs exhibited evidence of concurrent ADH. One dog showed no clinical suspicion of hypercortisolism preoperatively yet experienced poor postoperative recovery; a postoperative ACTH stimulation test then confirmed hypocortisolism. This dog also had an adrenocortical neoplasia on histopathology. A second dog tested negative for hypercortisolism via an IV low‐dose dexamethasone suppression test; however, its endogenous ACTH was suppressed, and postoperative ACTH stimulation confirmed a diagnosis of hypocortisolism. Histopathological examination subsequently revealed an adrenocortical tumour in addition to a PCC. The last dog underwent a urinary corticoid: creatinine ratio (UCCR) combined with a high‐dose dexamethasone suppression test; although the UCCRs did not exceed the cutoff for hypercortisolism, inadequate suppression and suppressed endogenous ACTH were observed. However, insufficient cortical tissue on the histopathological slides prevented a definitive assessment of the presence of an adrenocortical tumour.

### 3.3 Histopathological Parameters and Ki67 PI

All dogs that underwent adrenalectomy (*n* = 41) were included in the intra‐ and inter‐observer agreement analyses. Three histopathological features—necrosis, tumour cell spindling, and extension into adipose tissue—consistently demonstrated agreement scores above 0.4 for both intra‐ and inter‐observer comparisons (Table 2). The Ki67 PI showed excellent inter‐observer agreement (0.95; 95% CI 0.91–0.98; *p* < 0.001). Intra‐observer agreement could not be calculated for one observer, who scored each tumour only once, but the other observer scored 19 tumours twice, yielding an intra‐observer agreement of 0.99 (95% CI 0.99–1.0; *p* < 0.001).

**TABLE 2:** Intra‐ and interobserver agreement scores.

Parameter	Intra‐observer 1	Intra‐observer 2	Inter‐observer (score 1 vs. 1)	Inter‐observer (score 2 vs. 2)
Growth pattern	0.22 *p =* 0.055	0.044 *p =* 0.56	−0.074 *p =* 0.26	0.082 *p =* 0.17
**Necrosis**	**0.53** ***p <* 0.001**	**0.68** ***p <* 0.001**	**0.47** ***p =* 0.0015**	**0.40** ***p =* 0.0013**
Cellularity	0.29 *p =* 0.027	0.79 *p <* 0.001	−0.9 *p =* 0.20	0.13 *p =* 0.092
Cellular monotony	0.30 *p =* 0.0020	0.27 *p =* 0.041	0.18 *p =* 0.016	0.006 *p =* 0.90
**Tumour cell spindling**	**0.55** ***p <* 0.001**	**0.84** ***p <* 0.001**	**0.53** ***p <* 0.001**	**0.55** ***p <* 0.001**
Atypical mitotic figure(s)	0.29 *p =* 0.036	N/A	N/A	N/A
**Extension into adipose tissue**	**0.63** ***p <* 0.001**	**0.66** ***p <* 0.001**	**0.48** ***p <* 0.001**	**0.46** ***p <* 0.001**
Vascular invasion	0.68 *p <* 0.001	0.47 *p =* 0.0024	0.26 *p <* 0.001	0.079 *p =* 0.57
Capsular invasion	0.32 *p =* 0.035	0.61 *p <* 0.001	0.14 *p =* 0.30	0.21 *p =* 0.070
Nuclear pleomorphism	0.44 *p <* 0.001	0.40 *p <* 0.001	0.30 *p =* 0.006	0.21 *p =* 0.036

*Note*: Cohen's kappa coefficients (with corresponding *p* values) were used to evaluate agreement. N/A, not available; indicates that agreement could not be calculated because one or more variables were constant (e.g., one pathologist scored all patients as 0). Parameters included in the survival analysis are highlighted in bold.

### 3.4 Survival Analyses in Long‐Term Survivor Cohort (*n* = 33)

For the survival analyses, eight dogs that died within 2 weeks postoperatively were excluded to minimise the impact of surgery or anaesthesia‐related complications, leaving 33 dogs. Within this cohort, four dogs experienced tumour‐related events and 29 were censored. Detailed descriptions of the four tumour‐related events are provided in Supporting Information. Since more than half of the cases were still alive at the end of follow‐up, the median survival time was not reached, while the mean overall survival was 2456 days (95% CI: 2042–2871 days), and the median follow‐up time was 804 days. Only parameters with agreement scores ≥ 0.40 (necrosis, tumour cell spindling, extension into adipose tissue, and Ki67 PI) were included in the survival analyses. In univariate analyses, the three histopathological parameters did not meet the *p* ≤ 0.2 threshold in the Kaplan–Meier analyses, while Ki67 PI demonstrated a *P* value of 0.053 in univariate Cox regression. Although Ki67 PI was not significant at *p* < 0.05, its hazard ratio (HR = 1.15, 95% CI 0.998–1.324) suggests a 15% increase in risk of death per unit increase in Ki67 PI. As no other factors qualified for inclusion, a multivariate analysis was not performed. In addition, tumour size (maximum diameter on diagnostic imaging) was evaluated, but no significant association with outcome was found (*p* = 0.69).

### 3.5 Ki67 PI Cutoff Determination

**FIGURE 4:**
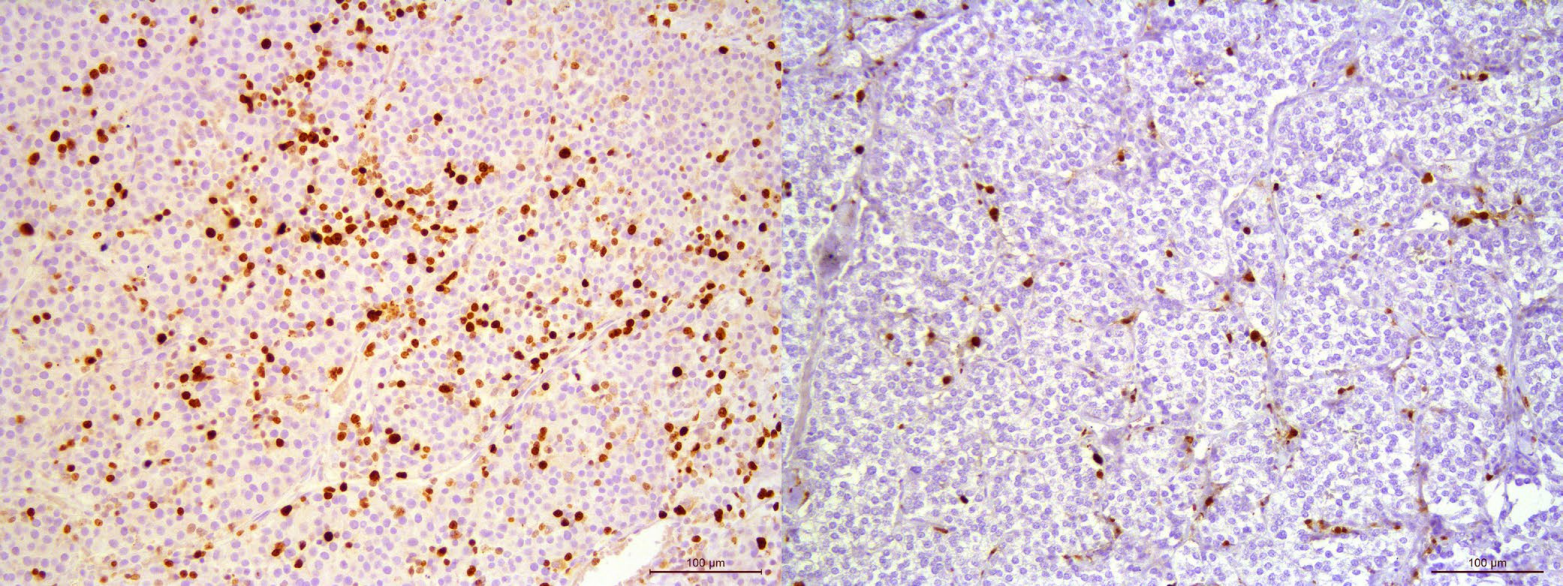
Ki67 proliferation index. Left panel: Tumour exhibiting a high Ki67 PI index (22%). Right panel: Tumour exhibiting a low Ki67 PI index (3.6%). Immunohistochemical staining for Ki67 using DAB as chromogen and counterstained with haematoxylin.

**FIGURE 5:**
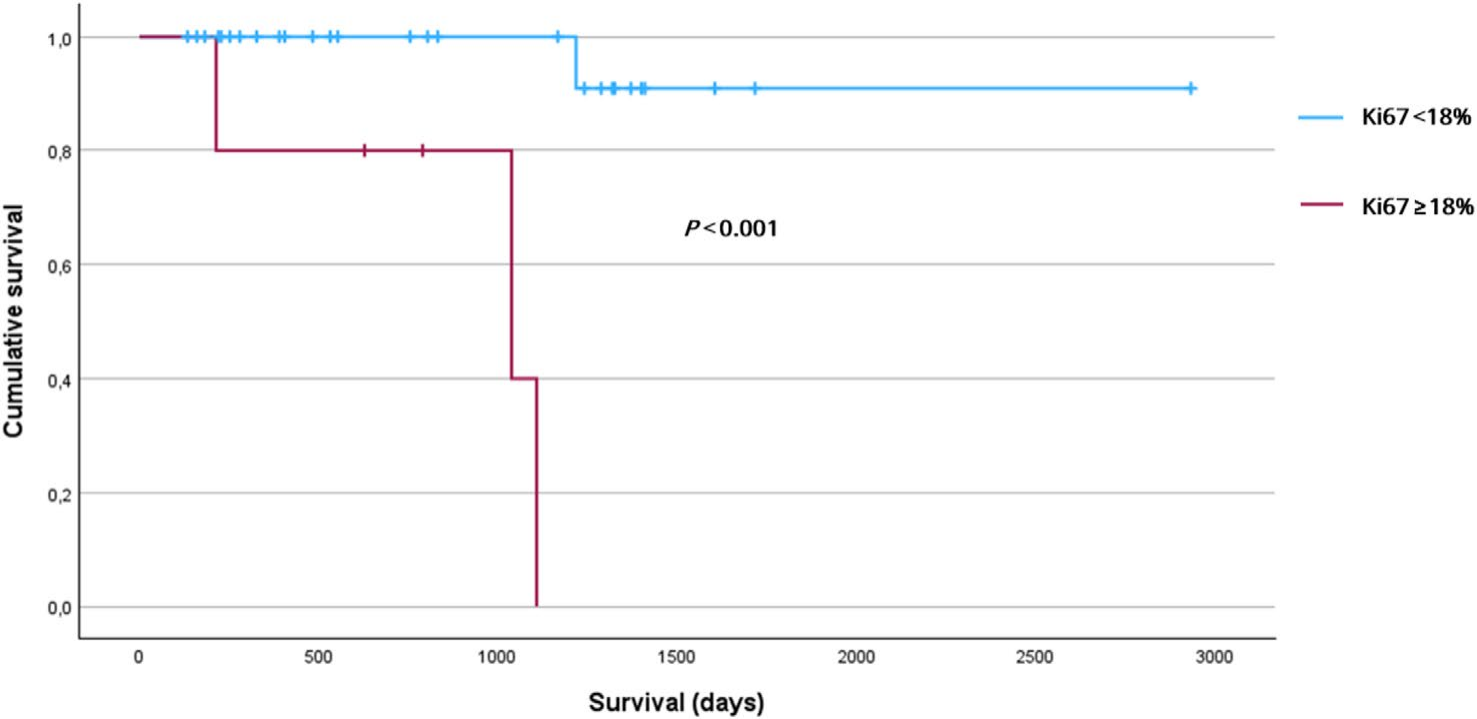
Survival stratified according to Ki67 PI cutoff of 18% using Kaplan–Meier analysis. Censored dogs are indicated as tick marks. The *p* value indicates the significance of the difference between Ki67 PI ≥ 18% and Ki67 PI < 18% as calculated with the log‐rank test.

To further explore Ki67 PI's prognostic value, we conducted an ROC analysis to determine whether a specific Ki67 PI threshold could better distinguish event versus non‐event outcomes in these 33 dogs. The area under the curve was 0.784, indicating moderate discriminatory ability. Among the tested cutoffs, a Ki67 PI of 18% provided the highest Youden's Index (0.681), with a sensitivity of 75% and a specificity of 93%. Consequently, we recoded Ki67 PI into two categories: < 18% (‘low’) versus ≥ 18% (‘high’). Kaplan–Meier analysis showed a significant difference in survival between the two groups (*p* < 0.001; Figures 4 and 5), although Cox regression could not be fitted due to non‐convergence of the coefficients. Therefore, we rely on the log‐rank result, concluding that a Ki67 PI ≥ 18% is associated with significantly worse survival.

### 3.6 Composite Histopathological Score

A composite histopathological score (range 0–4) was created by summing four binary parameters: necrosis, cell spindling, extension into adipose tissue, and Ki67 PI ≥ 18%. The score distribution was skewed, with 18 dogs scoring 0, 13 scoring 1, one scoring 2, none scoring 3, and one scoring 4. In a univariate Cox regression, the composite score approached but did not reach significance (*p* = 0.056), with a hazard ratio of 2.80 (95% CI: 0.975–8.028), suggesting a 2.8‐fold increase in the risk of death per unit increase in composite score. An overview of the event status and the individual components of the composite histopathological score for each dog is provided in Table S1.

## 4 Discussion

In this retrospective study, we investigated whether histopathological parameters—derived from human scoring systems for PCC prognostication—in addition to the immunohistochemical marker Ki67 PI, could be reliably assessed and used to predict survival in dogs after adrenalectomy for PCC. Our findings indicate that only three parameters (necrosis, tumour cell spindling, and extension into adipose tissue) achieved sufficient inter‐ and intra‐observer agreement (≥ 0.40) for inclusion in survival analyses, while Ki67 PI exhibited excellent reproducibility (agreement scores ≥ 0.95). The substantial variability observed in the remaining parameters suggests that comprehensive scoring systems such as GAPP or PASS, as currently applied, are not reliably transferable to canine PCC.

To develop a prognostic tool, we first established a composite histopathological score by summing the three reproducible parameters together with a dichotomised Ki67 PI (using an optimal cutoff of 18%). This approach was based on PASS and GAPP, which also use a total score derived from multiple parameters. We limited this to parameters with acceptable reproducibility. Although the composite score ranged from 0 to 4, its distribution was highly skewed, and in our long‐term survivor cohort—with a limited number of events—the Ki67 PI cutoff alone provided adequate prognostic stratification. Kaplan–Meier analysis demonstrated significantly poorer survival in dogs with a high Ki67 PI (≥ 18%), and ROC analysis demonstrated moderate discriminatory ability for this cutoff. Although the composite score showed a borderline association with outcome in Cox regression (*p* = 0.056; HR = 2.80), its added complexity and the limited contribution of the additional histopathological features suggest that, for this cohort, Ki67 PI as a standalone marker may be more reliable and clinically applicable for prognostication in canine PCC. Nonetheless, it remains possible that in a larger cohort with more events, the composite score might reveal additional prognostic value over Ki67 PI alone, warranting further investigation.

The relatively low to moderate observer agreement for several histopathological parameters underscores the subjective nature of these morphological assessments and highlights the need for a more unambiguous definition of individual parameters. The addition of various histochemical stains, such as Masson's trichrome or Gordon and Sweet's reticulin stain, might improve the evaluation of specific features, such as growth pattern. While intra‐observer reproducibility was generally higher than interobserver reproducibility, significant discrepancies between initial and repeat evaluations were noted for many parameters. Similar challenges have been reported with the PASS in human studies. Although PASS was initially proposed to differentiate tumours with aggressive behaviour from benign ones, a subsequent study revealed significant inter‐ and intra‐observer variation among experienced endocrine pathologists, leading to recommendations against its routine use for clinical prognostication [16]. In addition, another study showed low‐moderate interrater reliability for PASS [15]. In the current study, we therefore deliberately restricted our analyses to parameters with agreement scores ≥ 0.40 to mitigate this limitation. With respect to the grading of histopathological parameters and their association with clinical outcome, it is important to note that only a portion of each tumour was submitted for histopathological evaluation. Given that tumours are often heterogeneous, sampling bias could affect both qualitative parameters—and to a lesser extent, quantitative ones. Moreover, because PCCs are generally friable, they are prone to artifactual dispersion of individual or clustered neoplastic cells, which may falsely mimic vascular invasion.

The low number of events in our study—reflecting a generally favourable long‐term outcome following adrenalectomy for PCC—necessitates cautious interpretation of survival analyses. While our study identified significant prognostic information based on the Ki67 PI, the limited sample size and retrospective design may have introduced selection bias and incomplete data capture. Moreover, because no post‐mortem examinations were performed, we could not definitively confirm the cause of death in any case. Especially in the one case where a tumour‐related cause could not be excluded and was conservatively classified as an event, this introduces an element of uncertainty that we acknowledge as a limitation of the study. In addition, the Ki67 PI cutoff identified in our cohort may not be directly applicable to other laboratories, given potential variability in staining protocols and scoring methods. External validation is therefore recommended.

Existing data in literature on metastasis and recurrence following adrenalectomy for canine PCC is variable, largely due to differences in study populations and follow‐up duration. Moreover, the presence of concurrent neoplasms further complicates these assessments. Two studies have reported suspected or confirmed metastatic or recurrent disease in 18.5% and 21% of dogs with PCC, both with median follow‐up times of 16 months [4, 5], whereas others—with median follow‐up times ranging from 9 months to almost 1.5 years—have observed no such events [2, 9, 22]. Reported times to detection of recurrence or metastasis also vary widely. For example, one study documented recurrences between 75 and 1653 days after surgery, with metastases occurring at a mean of 348 ± 266 days postoperatively [5]. In comparison, definitive PCC‐related events (i.e., death due to recurrence or metastasis) were observed in 3 of 33 dogs (9%) in our study, with these dogs dying between 1038 and 1218 days postoperatively. In each of these cases, clinical signs had been evident several months beforehand—either as early signs later confirmed as metastasis/recurrence or as clear evidence of metastasis/recurrence. In one additional case, a PCC‐related event could not be definitively ruled out, and the patient was therefore counted as having experienced an event (death occurring 214 days postoperatively). These findings suggest that while some dogs may develop metastasis or recurrence relatively early, others experience these events much later, emphasising the need for long‐term follow‐up in this patient population. In addition, future studies incorporating molecular analyses such as whole‐genome sequencing may prove crucial in elucidating why certain dogs develop metastases and experience poorer outcomes.

Mutations in the succinate dehydrogenase subunit B (*SDHB*) gene have been associated with high metastatic risk in human PCC, with reported rates ranging between 35% and 75% [23]. SDHB IHC is used in the Modified GAPP to help identify these tumours with higher metastatic potential [24]. However, in dogs, the clinical relevance of *SDHB* mutations remains unclear despite reports of their occurrence [25, 26]. One study evaluated SDHB IHC in canine PCCs, but these findings were not validated by sequencing techniques [27]. Our own attempts to optimise SDHB staining were unsuccessful, echoing other reports that question its reliability in canine samples [25]. Consequently, SDHB IHC was not included in our study.

Immunohistochemistry for CHGA and SYP was used to confirm the diagnosis of PCC and was sometimes critical in differentiating these tumours from adrenocortical tumours. Although a subset of dogs exhibited a concurrent cortisol‐secreting adrenocortical tumour, the potential impact of this comorbidity on survival outcomes appears minimal in our study population, given that three of the four affected dogs were alive at the study endpoint, and one dog was euthanised 1326 days after surgery for reasons unrelated to adrenal disease. Additionally, two dogs in the cohort received systemic therapy (toceranib phosphate or capecitabine) following surgery. While such treatments may influence disease progression, the limited number of treated cases precludes meaningful conclusions regarding their effect on survival in this study.

In summary, our findings indicate that while a composite histopathological score integrating Ki67 PI, necrosis, tumour cell spindling, and extension into adipose tissue may offer comprehensive prognostic insight, the dichotomised Ki67 PI alone appears to be a reliable and practical predictor of long‐term outcome following adrenalectomy for canine PCC. Importantly, our data show that dogs undergoing adrenalectomy for PCC and surviving the immediate perioperative period generally exhibit a favourable prognosis. Nevertheless, a subset of patients still develops recurrence and/or metastasis. This suggests that dogs identified as having a poorer prognostic profile might benefit from more intensive screening for recurrence or metastasis, potentially enabling earlier intervention with additional treatment strategies. Future studies with larger, prospective cohorts and standardised scoring protocols are warranted to refine prognostication in canine PCC and ultimately guide postoperative management.

## Disclosure

The authors have no disclaimers to declare. Ethical approval was not required, as tissue samples were obtained from client‐owned dogs with naturally occurring phaeochromocytoma, collected postoperatively for curative purposes. Informed owner consent for the use of tissues in research was obtained in all cases.

## Conflicts of Interest

The authors declare no conflicts of interest.

## Supporting information


**Supplementary File 1.** Detailed case descriptions of tumour‐related events.

**Table S1** vco70021‐sup‐0002‐TableS1.xlsx

## Data Availability

The data that support the findings of this study are available from the corresponding author upon reasonable request.
