# Supplementary material for: Prognostic Value of a Histopathological Scoring System and the Ki67 Proliferation Index in Dogs With Phaeochromocytoma
Source: Vet Comp Oncol. 2025 Sep 3;24(1):11–20. doi: 10.1111/vco.70021 (PMC12875748; doi:10.1111/vco.70021)
Supplement: Supplementary file 1 — Detailed case descriptions of tumour‐related events. [file VCO-24-11-s002.docx]

**Supplementary File 1**. Detailed case descriptions of tumour-related events.

The first dog developed polyuria/polydipsia and weight loss approximately 2.5 years post-adrenalectomy, followed by weakness and vomiting. CT imaging revealed a large, poorly defined, and irregular soft tissue mass (40 x 29 x 41 mm) centered around the region of the previously excised left adrenal gland (where surgical clips were visible), and located dorsally within the retroperitoneum. The mass showed mild heterogeneous contrast enhancement and invaded the caudal vena cava, causing complete distortion of its normal architecture over a long segment, extending cranially towards the hepatic portion. Bilateral mass extension into the renal veins was also noted, resulting in vascular dilation, loss of pyelic architecture, and suspected early obstruction. Multiple regional lymph nodes (including para-aortic, renal, portocaval, jejunal, and pancreaticoduodenal nodes) were markedly enlarged, rounded, and heterogeneously enhancing. Both kidneys exhibited multiple, ill-defined nodules that disrupted the normal renal architecture and distorted the renal contours. The most prominent lesion, located in the caudal pole of the left kidney, measured approximately 3 cm and caused severe deformation of the kidney. The right adrenal gland could not be identified separately. Based on these findings, the disease was considered highly consistent with extensive local recurrence and metastasis of PCC. The dog was euthanised shortly thereafter.

The second dog, which had previously undergone adrenalectomy for a right-sided PCC, was re-evaluated approximately three years later due to clinical deterioration. CT imaging revealed a large, irregular, and heterogeneously enhancing mass in the region of the right adrenal gland (21.5 × 20.2 × 31 mm), consistent with local tumour recurrence. Additionally, a large lobulated hepatic lesion (57 × 36 × 48 mm) with heterogeneous contrast uptake was observed, alongside a smaller, well-enhancing peritoneal nodule. The lungs showed at least twenty, well-defined, round nodules of varying size (up to 8.6 mm), bilaterally distributed with peripheral predominance, highly suggestive of pulmonary metastases. Cytology of the hepatic mass was consistent with a neuroendocrine tumour. Based on these findings, the disease was considered highly consistent with widespread metastatic recurrence of PCC. The dog died spontaneously four months later.

The third dog was re-evaluated approximately 2.5 years after adrenalectomy due to recurrent clinical signs. At that time, the plasma normetanephrine concentration was markedly elevated (51.52 nmol/L). Follow-up imaging confirmed local regrowth at the site of the previously excised adrenal gland and metastatic lesions in the liver. The dog was euthanised five months later. Based on the clinical course and diagnostic findings, the disease was considered consistent with metastatic recurrence of PCC.

The fourth dog died 214 days postoperatively. The exact cause of death could not be confirmed, as the owner declined further diagnostic investigation. However, a tumour-related cause could not be confidently excluded, and the case was therefore classified as an event.
